# Titin as a potential novel therapeutic target in colorectal cancer

**DOI:** 10.1111/jcmm.17866

**Published:** 2023-07-27

**Authors:** Hongyun Wei, Keyu Ren, Qian Zhang, Yanchun Jin, Bin Cao, Zibin Tian, Tao Mao, Linlin Ren

**Affiliations:** ^1^ Department of Gastroenterology Affiliated Hospital of Qingdao University Qingdao China

**Keywords:** colorectal cancer, metastasis, novel target, proliferation, titin

## Abstract

Colorectal cancer (CRC) is identified as a primary cause of death around the world. The current chemotherapies are not cost‐effective. Therefore, finding novel potential therapeutic target is urgent. Titin (TTN) is a muscle protein that is critical in hypertrophic cardiomyopathy. However, its role in CRC is not well understood. The study focused on exploring the possible role of TTN in CRC carcinogenesis. TTN mRNA and protein expression levels presented an obvious downregulation in CRC tissue samples, relative to normal control (*p* < 0.05). TTN expression significantly correlated with the clinical stage (normal vs. Stage 1, *p* < 0.05; normal vs. Stage 4, *p* < 0.05), node metastasis (normal vs. N1, *p* < 0.05; N1 vs. N2, *p* < 0.05), histological type (normal vs. adenocarcinoma, *p* < 0.05), race (Caucasian vs. Asian, *p* < 0.05; African‐American vs. Asian, *p* < 0.05) and TP53 mutation (normal vs. TP53 mutation, *p* < 0.05), considering The Cancer Genome Atlas database. However, for patients who had higher TTN expression, the overall survival was remarkably shorter than patients who had low TTN expression. Furthermore, TTN was lowly expressed in four CRC cell lines. TTN overexpression facilitated CRC cells in terms of the proliferation, metastasis and invasion. Based on gene set enrichment analysis, the ERB pathway might be responsible for TTN‐related CRC. Besides, TTN was involved in the response to azacitidine. Overall, TTN might serve as a potential novel therapeutic target for treating and overcoming chemotherapy resistance in CRC.

## 1 INTRODUCTION

According to recent cancer statistics, cancer is still the primary cause of death globally.^1^ Colorectal cancer (CRC) ranks fourth in causing cancer‐related death around the world.^2^, ^3^, ^4^ Multidrug chemotherapy is often recommended as a first‐line treatment approach for CRC.^4^, ^5^, ^6^ However, first‐line treatment with chemotherapy is not cost‐effective and does not improve long‐term overall survival (OS).^6^, ^7^, ^8^, ^9^ In addition, although immunotherapies such as anti‐PD‐1 antibodies have been developed for CRC treatment,^10^ only a few patients show durable responses.^10^, ^11^ Therefore, exploring potential therapeutic targets for CRC treatment is critical.

Titin is a muscle protein that is encoded by the *TTN* gene. TTN contains an N‐terminal I‐band and a C‐terminal A‐band. The A‐band possesses kinase activity and participates in active contraction. The I‐band contains two regions of the tandem immunoglobulin domain, which can bind proteins implicated in protein quality control pathways.^12^ TTN is mainly expressed in skeletal and heart muscles. TTN has been shown to participate in the process of familial hypertrophic cardiomyopathy^13^ and autoimmune disease scleroderma.^14^ Besides, it is one of the most frequently mutated genes in solid tumours such as gastric cancer.^15^, ^16^ Patients with a higher rate of TTN mutation had a poor prognosis for OS, but a positive response to immune checkpoint blockade therapies,^17^ indicating the potential role of TTN in solid tumours. The adverse impact of TTN on the prognosis of solid tumours is largely attributed to its association with chemotherapy resistance. In a study on hepatocellular carcinoma, the *TTN* gene‐related lncRNA *TTN‐AS1* was found to regulate the transcriptional activity of relevant genes by targeting miR‐16‐5p and the tumour suppressor gene *PTEN*, resulting in decreased sensitivity of tumour cells to sorafenib. The resistance significantly diminished upon depletion of *TTN‐AS1*.^18^ Similarly, studies on other solid tumour types such as breast cancer,^19^ ovarian cancer and lung cancer^20^ have also revealed an association between low TTN expression and chemotherapy resistance. However, these studies are still in the early stages, and the exact mechanisms and interactions between TTN and chemotherapy resistance need further investigation, particularly in different tumour types and individuals. Nevertheless, researchers have not clearly explored the impact of TTN on CRC development. Our study aimed at clarifying such impact, as well as providing a potential novel therapeutic target specific to CRC.

## 2 MATERIALS AND METHODS

### 2.1 Sample collection

CRC tissues and normal controls came from two cohorts receiving surgery at the Affiliated Hospital of Qingdao University. Samples of 19 CRC patients and 19 adjacent normal controls collected between 2016 and 2018 were included in Cohort 1. Samples of 32 CRC patients and 32 normal controls collected between 2017 and 2020 were included in Cohort 2. Clinicopathological features, including gender, age, TNM stage, tumour size and degree of differentiation, were recorded for the study cohorts. Our study has obtained the approval of the Ethics Committee of the Affiliated Hospital of Qingdao University, and obtained all participants' informed consent.

### 2.2 Cells lines and cell culture

HCT116, LoVo, HT‐29 and SW620 CRC cell lines were provided by the Cell Bank of the Chinese Academic of Sciences. HCT116, LoVo, HT‐29 and SW620 CRC cell lines were cultured in DMEM/F‐12 (Hyclone) with 10% FBS (Gibco) and 1% penicillin–streptomycin (Hyclone). Mycoplasma testing has been carried out for the cell lines used.

### 2.3 Immunohistochemistry (IHC)

Twenty percent formaldehyde‐fixed tissue samples underwent paraffin embedding, followed by being cut into thin sections (5 μm) and rehydrated. Then heat‐induced antigen retrieval was conducted in citrate buffer or Tris/EDTA buffer. Tissue sections received 10 min of blocking in 3% hydrogen peroxide (H_2_O_2_) at room temperature (RT). The sections underwent one night of incubation at 4°C using rabbit polyclonal antibody against TTN (1:100, bs9861‐R; Bioss). Later, the sections underwent the treatment of horseradish peroxidase conjugated anti‐rabbit IgG antibody (1:500) for 30 min at RT. The non‐specific binding and antibody detection were blocked by the DAB Detection kit. Tissue sections then received haematoxylin counterstaining. A light microscope served for the detection of immunostained sections. Image‐Pro Plus (version 6.0) served for the semiquantitative analysis. In addition, The Cancer Genome Atlas (TCGA) database assisted in validating the association of TTN expression with CRC clinical features.

### 2.4 RNA isolation and quantitative PCR (qPCR)

TRIzol reagent (Invitrogen) served for extracting the total RNA from cell lines and colon samples. PrimerScript TM RT reagent Kit with gDNA Eraser (TAKARA) served for the synthesis of first‐strand cDNA following the protocol of the manufacturer. The primers for TTN included, F: 5′‐GCGTAAGACTCAGGCATCCA‐3′ and R: 5′‐AGTAGAGGTTGTCAGCGTTGT‐3′. We conducted qPCR under the conditions of 30 min of predenaturation at 95°C, 5 s of 40 cycles of denaturation at 95°C, annealing and 34 s of elongation at 60°C. Target gene mRNA expression was amplified and analysed with ABI Prism 7300 SDS Software using GAPDH as an internal standard. The 2^−ΔΔCT^ method served for calculating the relative transcripts. We repeated all experiments for three times. The primer for GAPDH was forward: 5′‐GTCTTCACCACCATGGAGAA‐3′; reverse: 5′‐TAAGCAGTTGGTGGTGCAG‐3′.

### 2.5 Overexpression of TTN

Guide RNA (gRNA) for site‐specific gene editing mediated by CRISPRa was used to trans‐activate TTN expression. The plasmids lenti dCAS‐VP64‐puro, lenti sgRNA(TTN)‐MPH‐Blast were used for CRISPR‐Cas9 activation. Subcloning PCR‐amplified Single‐guide RNA (sgRNA) of TTN was sub‐cloned into the H7284 plenti‐U6‐gRNA‐2x(wt+f6)MS2‐CMV‐MCP‐P65‐HSF1‐IRES‐Blast vector (OBiO Technology) using the forward primer: 5′‐CCGGGTATGTGACACTACCAAAT‐3′ and reverse primer: 5′‐AAACATTTGGTAGTGTCACATAC‐3′, so as to generate the TTN expression construct. The LoVo cell line was transduced by lenti dCAS‐VP64‐puro and lenti sgRNA(TTN)‐MPH‐Blast. Stable cell lines with overexpressed TTN were selected using puromycin (2 μg/mL) and blasticidin (10 μg/mL). qPCR served for the detection of the mRNA expression level of TTN after transfection.

### 2.6 Cell viability assay

Cell counting kit‐8 (CCK‐8) served for detecting the cell viability. We plated LoVo, LoVo‐H7281+vector and LoVo‐H7281 cells in 96‐well plates (2 × 10^3^ cells/well). 0, 24, 48, 72 and 96 h of treatment was followed by the addition of 10 μL CCK‐8 solution into each well. The plates received 2 h of incubation at 37°C. An automatic microplate reader (Spark 10M, TECAN) served for reading the absorbance at 450 nm.

### 2.7 Migration and invasion assay

A wound‐healing assay assisted in assessing CRC cell migration ability. Cells were seeded in a 6‐well plate and cultured overnight. A 200 μL pipette tin was employed to scratch a straight line, which was then cultured in a fresh medium. Migrated cells were detected with a microscope (Leica) at 0, 24, 48, 72, 96 and 120 h. Image‐pro plus 6.0 software was used for the analysis of the images. Next, a transwell assay served for the evaluation of the cell invasion. Briefly, cells seeded in a matrigel invasion chamber (Corning) received 48 h of culturing at 37°C. Invaded cells received crystal violet staining (Beyotime). A microscope illustrated related images.

### 2.8 Statistical analyses

Data were in the form of mean ± standard deviation. SPSS 20 (IBM) served for all statistical analyses. Student's *t*‐test or two‐way anova tests served for comparing the means between two or three groups. The Kaplan–Meier method assisted in drawing survival curves. A *p* < 0.05 reported statistical significance.

## 3 RESULTS

### 3.1 Downregulation of TTN in CRC tissues and cells

**FIGURE 1:**
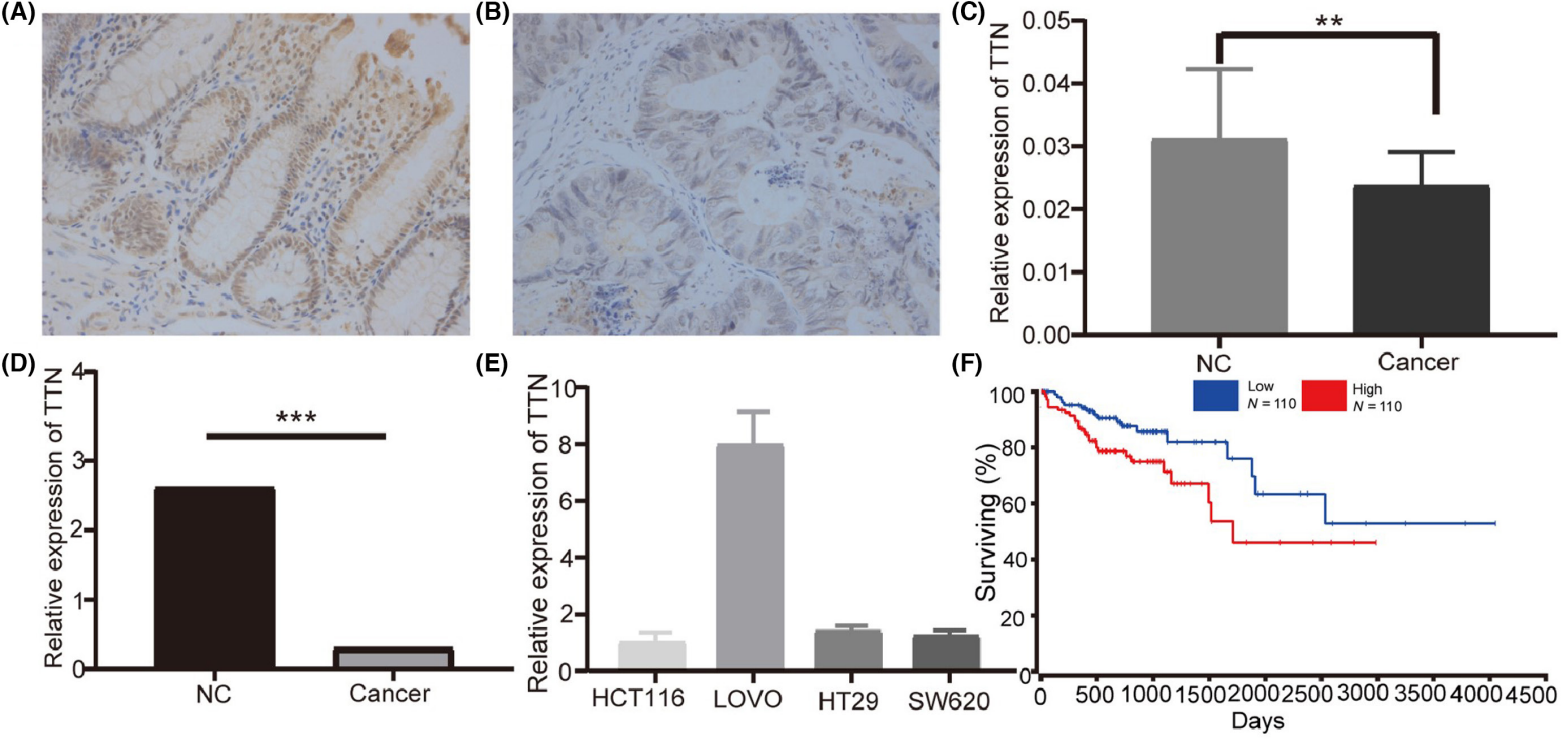
Expression level of titin (TTN) in colorectal cancer tissues and cells. Immunohistochemistry (IHC) analysis demonstrated that TTN expression was downregulated in colorectal tumour tissues (B) compared to control (A). (C) The statistics between the tumour tissue and the control group of the IHC experiment results. (D) Analysis of TTN mRNA expression using RT‐PCR (***p < 0.01). (E) Basal expression of TTN in HCT116, LoVo, HT29 and SW620 cells. (F) Kaplan–Meier survival curves for colorectal cancer patients stratified by high and low expression of TTN.

For investigating TTN expression profile in CRC tissues, we explored its expression in CRC and adjacent tissues using IHC. The basic clinical features are shown in Table 1. We found that TTN was downregulated in CRC samples (*n* = 19) compared to adjacent normal tissues (*n* = 19). Representative images of the IHC experiment are shown in Figure 1A,B. Additionally, we performed intergroup statistical analysis of the expression levels (*p* = 0.008) (Figure 1C). To verify the mRNA expression level of TTN, we also performed RT‐PCR in CRC tissues and normal controls (*n* = 32). Similarly, TTN presented an obvious downregulation in CRC tissues, relative to normal controls (2.61 ± 3.82 vs. 0.31 ± 0.41, *p* < 0.01) (Figure 1D). TTN expression was also assessed in the four CRC cells (HCT‐116, LoVo, HT29 and SW620) using RT‐PCR. A high cycle threshold (CT) value (CT > 15) was observed in the four cell lines (Figure 1E). Accordingly, TTN presented a downregulation in human CRC.

**TABLE 1:** The characterizes of patients from Cohort 1.

TTN expression group	High expression	Low expression
Cases	9 (47.4%)	10 (63.2%)
Age (years)		
≥60	7 (77.8%)	5 (50%)
<60	2 (22.2%)	5 (50%)
Gender		
Male	6 (66.7%)	4 (40%)
Female	3 (33.3%)	6 (60%)
Stage		
I–II	4 (44.4%)	5 (50%)
III–IV	5 (55.6%)	5 (50%)
Tumour size		
<4	3 (33.3%)	3 (30%)
≥4	6 (66.7%)	7 (70%)
Differentiation		
Poor	0 (0%)	1 (10%)
Well/moderate	9 (100%)	9 (90%)
Node metastasis		
Absent	9 (100%)	10 (100%)
Present	0 (0%)	0 (0%)
Expression of Ki67		
Low	4 (44.4%)	6 (60%)
High	5 (55.6%)	4 (40%)

### 3.2 Association between TTN downregulation and advanced clinicopathological features and poor OS in CRC

**FIGURE 2:**
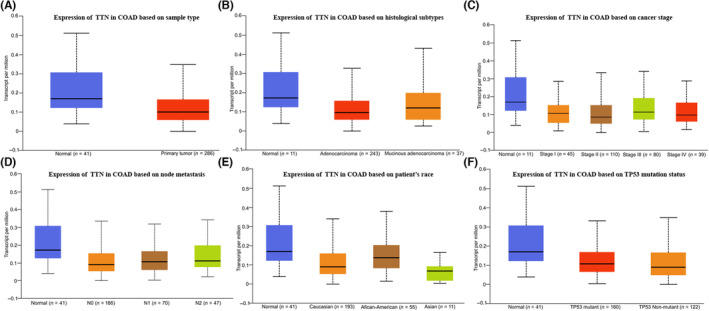
Downregulation of titin (TTN) is associated with advanced clinicopathological features of colorectal cancer based on the The Cancer Genome Atlas database. Expression level (A), histological type (B), clinical stage (C), node metastasis (D), race (E) and TP53 mutation (F).

Based on the TCGA database, TTN expression was significantly associated with the clinical stage (normal vs. Stage 1, *p* < 0.05; normal vs. Stage 4, *p* < 0.05), node metastasis (normal vs. N1, *p* < 0.05; N1 vs. N2, *p* < 0.05), histological type (normal vs. adenocarcinoma, *p* < 0.05), race (Caucasian vs. Asian, *p* < 0.05; African‐American vs. Asian, *p* < 0.05) and TP53 mutation (normal vs. TP53 mutation, *p* < 0.05) (Figure 2A–F). In summary, our findings demonstrated that TTN protein was downregulated in CRC tissue and patients with poorly differentiated CRC had remarkably lower TTN expression level. Next, we examined the impact of TTN on patients' OS using the Kaplan–Meier survival curves. For patients who had higher TTN expression levels, the OS was obviously shorter, relative to patients who had lower TTN expression, based on the TCGA database (*p* = 0.02) (Figure 1F).

### 3.3 Positive impact of TTN overexpression on proliferation, metastasis and invasion of CRC cells

**FIGURE 3:**
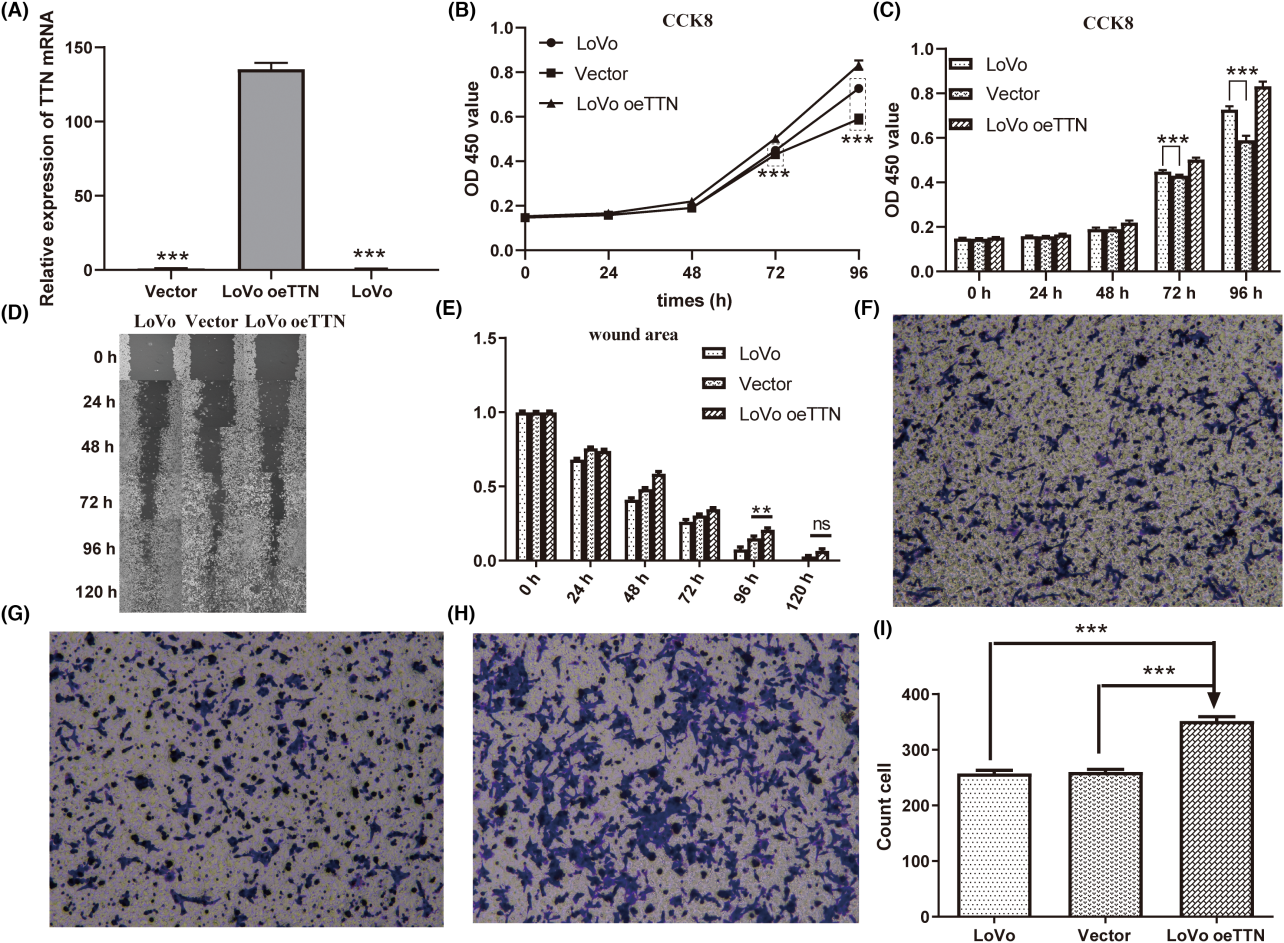
Overexpression of titin (TTN) promotes the proliferation, metastasis, and invasion of colorectal cancer cells. (A) Overexpression of TTN was verified using RT‐qPCR (***p < 0.001 vs. LoVo oeTTN). (B, C) Cell counting kit‐8 (CCK‐8) assays were performed after overexpression of TTN (***p < 0.001 vs. LoVo oeTTN). (D, E) Wound‐healing analysis was performed to investigate the effect of TTN on the motility of colorectal cancer cells (**p < 0.01 vs. LoVo oeTTN). (F–I) Transwell assays were used to determine the invasiveness of cells with blank LoVo cells (F), vector (G) and upregulated TTN (H) (***p < 0.01).

For evaluating the role of TTN in CRC tumorigenesis, TTN overexpression in LoVo cells was investigated. Effective overexpression of TTN in LoVo cells was detected using RT‐PCR, and LoVo CRC cells presented more obvious TTN overexpression, relative to vector control cells (*p* < 0.001) (Figure 3A). The CCK‐8 assays served for detecting cell viability. As the experiment time went by, the cell survival levels of the three groups all increased, and by 96 h after treatment, the cell survival levels of the LoVo oeTTN group with *TTN* overexpression were significantly higher than those of the control and vector groups at the same time (*p* < 0.001) (Figure 3B,C). This result suggested that after TTN was overexpressed, tumour cell proliferation in CRC could be significantly enhanced and the course of the cancer might be accelerated. A wound‐healing assay served for examining the effect of TTN on the motility of CRC cells, finding that the migration ability was not significantly increased in TTN‐overexpressed cells compared to vector control cells (Figure 3D,E). A transwell assay assisted in confirming the role of TTN in the invasion. Hence, overexpression of TTN promoted CRC cell invasion (Figure 3F–I).

### 3.4 Gene set enrichment analysis

**FIGURE 4:**
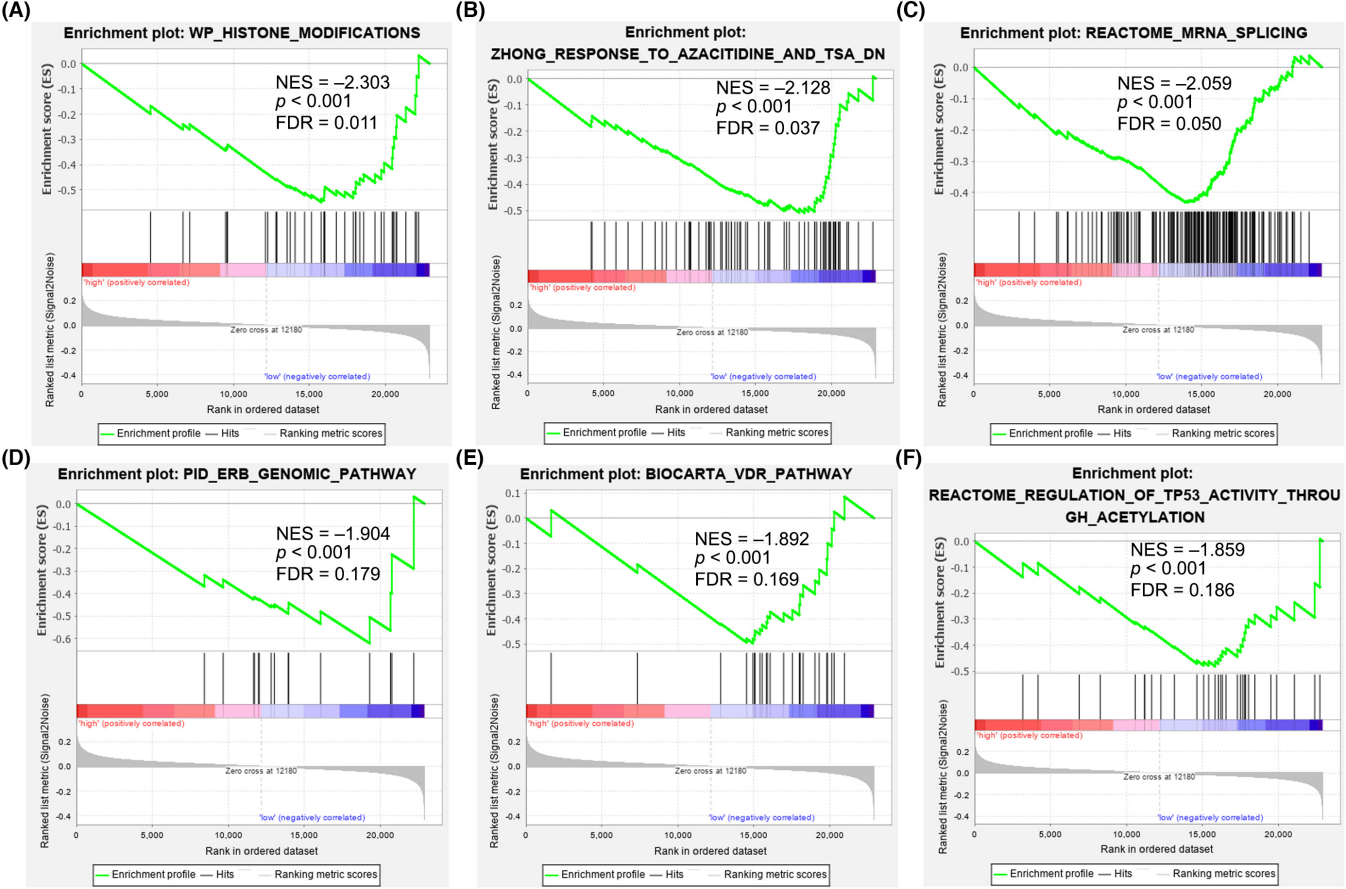
GSEA analysis. Six pathways, including histone modifications (A), response to azacitidine (B), mRNA splicing (C), ERB pathway (D), VDR pathway (E) and regulation of TP53 activity through acetylation (F), showed significant differential enrichment in titin (TTN) low expression phenotype based on normalized enrichment score (NES), nominal *p* value and false discovery rate value.

Gene set enrichment analysis (GSEA) served for analysing the underlying pathways of TTN involved in the development of CRC, finding that six pathways, namely, histone modifications, response to azacitidine, mRNA splicing, ERB pathway, VDR pathway, regulation of TP53 activity through acetylation, were significantly differentially enriched in low‐expressed TTN phenotype in terms of normalized enrichment score (NES), nominal *p* value, and te value (Figure 4A–F; Table 2). GSEA analysis of these six signalling pathways reported to be associated with CRC progression and chemotherapy resistance all showed downregulation (NES < −1) after TTN knockout compared with the control group. Overall, all these illustrate the potential role of TTN in CRC development and chemosensitivity.

**TABLE 2:** Gene sets enriched in phenotype low.

MSigDB collection	Gene set name	NES	NOM *p* value	FDR *q* value
c2.all.v7.2.symbols.gmt	Histone modifications	−2.30304	0	0.010794
	Response to azacitidine and TSA DN	−2.12813	0	0.036731
	Reactome mRNA splicing	−2.05878	0	0.050342
	ERB genomic pathway	−1.90392	0	0.179246
	Biocarta VDR pathway	−1.89216	0	0.169402
	Reactome regulation of TP53 activity through acetylation	−1.85885	0	0.185698

Abbreviations: FDR, false discovery rate; NES, normalized enrichment score; NOM, nominal.

## 4 DISCUSSION

Over the last decades, although various approaches have been employed in CRC treatment,^21^ there has been little progress in CRC prevention strategies.^4^, ^22^ Due to unsatisfactory outcomes of available chemotherapies, novel treatment strategies for CRC are needed urgently.^23^ The current study aimed at examining TTN‐CRC relationship, as well as proving a potential novel therapeutic target for CRC. IHC and qPCR results showed low expression of TTN in CRC tissues. In addition, TTN expression presented an obvious relevance to the clinical characteristics and CRC patients' OS.

Manipulating the expression of TTN in tumour cells revealed the positive impact of TTN overexpression on colon cancer cells in terms of the proliferation, invasion and metastasis, proving the involvement of TTN in colon cancer progression. Low TTN expression may indicate a poor prognosis. We further verified the impact of TTN expression on prognosis through survival analysis, finding the lower OS in group with high expression relative to group with low expression, which was consistent with cellular results.

However, the result of CRC sample based on IHC seemed contrasted with the functional experiments of the LoVo cell. The results from IHC showed TTN was downregulated in CRC tissue, while over‐expressed TTN promoted CRC cell proliferation and migration in vitro. The possible explanation for the above results is that TTN was mainly observed to express in inflammatory cells based on IHC results. While the normal colon tissue has higher proportion of inflammatory cells than CRC tissues, higher TTN expression in the normal tissues was showed in IHC results compared to tumour tissues. In fact, TTN might be a potential oncogene in the development of CRC. Therefore, in tissues with high tumour heterogeneity, single‐cell sequencing can be conducted to avoid the interference caused by inflammatory cells in the tumour microenvironment. These results also indicate the potential impact of inflammation on CRC development, which is consistent with previous studies.^24^, ^25^, ^26^ Accumulating evidence has shown that chronic bowel inflammation contributes to CRC initiation and progression.^27^, ^28^

In the GSEA analysis results, we identified six pathways that play important roles in the CRC carcinogenesis or chemoresistance. Histone modifications play a crucial role in regulating gene expression and chromatin structure. Aberrant histone modifications, such as histone methylation and acetylation, can lead to dysregulated gene expression patterns, including the activation of oncogenes and the silencing of tumour suppressor genes.^29^ These alterations contribute to the dysregulation of key signalling pathways involved in CRC pathogenesis. Altered histone modifications can affect the accessibility of DNA to azacitidine, potentially influencing its efficacy in reversing DNA methylation and restoring normal gene expression patterns in chemotherapy‐resistant CRC cells. In the context of chemotherapy resistance, histone modifications may impact the response to the DNA methyltransferase inhibitor including azacitidine used in CRC treatment.^30^ The mRNA splicing is a crucial process that removes introns and joins exons to generate mature mRNA molecules. Dysregulation of mRNA splicing has been implicated in various diseases, including cancer.^31^ In CRC, alterations in mRNA splicing patterns have been observed and associated with tumour progression and drug resistance. It has been reported that dysregulation of the ERB pathway and VDR pathway plays a promoting role in the progression and chemotherapy resistance of various cancers, including CRC.^32^, ^33^ This involves the survival and proliferation of tumour cells, drug efflux mechanisms within the tumour microenvironment and modulation of immune response regulation. TP53, also known as p53, is a well‐known tumour suppressor gene. Acetylation of TP53 enhances its transcriptional activity and promotes the expression of genes involved in tumour cell cycle arrest, DNA repair and tumour cell apoptosis.^34^ All of these pathways were downregulated in the GSEA enrichment analysis, implying that these positive signals for CRC progression and chemoresistance were abolished after TTN knockout, confirming previous conjectures and conclusions of present study.

Overall, the present study provides evidence for the role of TTN in CRC and confirms that inflammation remarkably impacts CRC development. Furthermore, GSEA was used to analyse the possible mechanisms of TTN‐induced colon cancer and found that the ERB pathway was associated with TTN in colon cancer based on bioinformatics analysis. Besides, TTN was also associated with response to azacitidine, indicating its potential role in improving chemotherapy resistance. To sum up, TTN critically impacts CRC occurrence and development through the ERB pathway and its high expression in cancer cells indicates a poor prognosis of colon cancer. Therefore, TTN might be a novel therapeutic target specific to CRC.

## 5 CONCLUSION

According to the results in the study, TTN may remarkably impact colon cancer process through the ERB pathway and high expression of TTN indicates a poor prognosis of colon cancer patients. Importantly, TTN can indicate the prognosis of CRC and serve as a new therapeutic target.

## AUTHOR CONTRIBUTIONS

**Hongyun Wei:** Formal analysis (lead); funding acquisition (lead); methodology (lead); project administration (lead); software (lead); writing – original draft (lead). **Keyu Ren:** Software (equal); writing – review and editing (supporting). **Qian Zhang:** Formal analysis (equal); methodology (equal); writing – original draft (supporting). **Yanchun Jin:** Writing – original draft (supporting). **Bin Cao:** Data curation (lead); validation (lead); writing – review and editing (equal). **Zibin Tian:** Conceptualization (equal); data curation (supporting); writing – review and editing (supporting). **Tao Mao:** Validation (equal); writing – review and editing (equal). **Linlin Ren:** Conceptualization (lead); data curation (supporting); funding acquisition (equal); project administration (equal); supervision (lead); validation (supporting); writing – review and editing (supporting).

## FUNDING INFORMATION

The work was completed with the support of the National Natural Science Foundation of China (No. 81602056, 82273393) and the Natural Science Foundation of Shandong Province (No. ZR2016HQ45, ZR2020LZL004) to Linlin Ren, Shandong medical and health science and technology development plan project (Grant number 202003030357) and Postdoctoral Science Foundation of China under Grant RZ2100002858 to Hongyun Wei.

## CONFLICT OF INTEREST STATEMENT

The authors declare no conflict of interest.

## Data Availability

The data supporting the findings of our study can be obtained from the corresponding author on reasonable request, and are openly available in TCGA at https://portal.gdc.cancer.gov/.
